# Effect of High-Dose vs Standard-Dose Vitamin D_3_ Supplementation on Body Composition among Patients with Advanced or Metastatic Colorectal Cancer: A Randomized Trial

**DOI:** 10.3390/cancers12113451

**Published:** 2020-11-20

**Authors:** Justin C. Brown, Michael H. Rosenthal, Chao Ma, Sui Zhang, Halla S. Nimeiri, Nadine J. McCleary, Thomas A. Abrams, Matthew B. Yurgelun, James M. Cleary, Douglas A. Rubinson, Deborah Schrag, Andrea J. Bullock, Jill Allen, Dan Zuckerman, Emily Chan, Jennifer A. Chan, Brian Wolpin, Michael Constantine, Douglas J. Weckstein, Meredith A. Faggen, Christian A. Thomas, Chryssanthi Kournioti, Chen Yuan, Hui Zheng, Bruce W. Hollis, Charles S. Fuchs, Kimmie Ng, Jeffrey A. Meyerhardt

**Affiliations:** 1 Pennington Biomedical Research Center, Baton Rouge, LA 70808, USA; 2 LSU Health Sciences Center, New Orleans School of Medicine, New Orleans, LA 70012, USA; 3 Stanley S. Scott Cancer Center, Louisiana State University Health Sciences Center, New Orleans, LA 70012, USA; 4 Dana-Farber Cancer Institute and Harvard Medical School, Boston, MA 02115, USA; Michael_Rosenthal@dfci.harvard.edu (M.H.R.); Chao_Ma@DFCI.HARVARD.EDU (C.M.); sui_zhang@jhu.edu (S.Z.); NJ_McCleary@DFCI.HARVARD.EDU (N.J.M.); Thomas_Abrams@DFCI.HARVARD.EDU (T.A.A.); Matthew_Yurgelun@dfci.harvard.edu (M.B.Y.); JCLEARY@PARTNERS.ORG (J.M.C.); douglas_rubinson@dfci.harvard.edu (D.A.R.); Deb_Schrag@dfci.harvard.edu (D.S.); JANG@PARTNERS.ORG (J.A.C.); Brian_Wolpin@dfci.harvard.edu (B.W.); chen_yuan@dfci.harvard.edu (C.Y.); kimmie_ng@dfci.harvard.edu (K.N.); Jeffrey_Meyerhardt@dfci.harvard.edu (J.A.M.); 5 Division of Hematology Oncology, Department of Medicine, Northwestern University, Chicago, IL 60611, USA; hnimeiri@nm.org; 6 Beth Israel Deaconess Medical Center, Boston, MA 02215, USA; abullock@bidmc.harvard.edu; 7 Massachusetts General Hospital, Boston, MA 02114, USA; jallen0@partners.org (J.A.); hzheng1@partners.org (H.Z.); 8 St Luke’s Mountain States Tumor Institute, Boise, ID 83712, USA; zuckermd@slhs.org; 9 Vanderbilt University Medical Center, Nashville, TN 37232, USA; emyychan@yahoo.com; 10 Dana-Farber at Milford Regional Medical Center, Milford, MA 01757, USA; Michael_Constantine@DFCI.HARVARD.EDU; 11 New Hampshire Oncology Hematology, Hookset, NH 03106, USA; D.Weckstein@nhoh.com; 12 Dana-Farber at South Shore Hospital, South Weymouth, MA 02190, USA; MEREDITH_FAGGEN@DFCI.HARVARD.EDU; 13 New England Cancer Specialists, Scarborough, ME 04074, USA; thomac@newecs.org; 14 Newton-Wellesley Hospital, Newton, MA 02462, USA; CKOURNIOTI@PARTNERS.ORG; 15 Department of Pediatrics, Department of Medicine, Medical University of South Carolina, Charleston, SC 29425, USA; hollisb@musc.edu; 16 Yale Cancer Center, New Haven, CT 06510, USA; charles.fuchs@yale.edu

**Keywords:** adipose tissue, colorectal neoplasms, cholecalciferol, mediation, prognosis, randomized, skeletal muscle

## Abstract

**Simple Summary:**

Skeletal muscle and adipose tissue express the vitamin D receptor and may be a mechanism through which vitamin D supplementation slows cancer progression and reduces cancer death. It is unknown if high-dose vitamin D_3_ impacts skeletal muscle and adipose tissue, as compared with standard-dose vitamin D_3_, in patients with advanced or metastatic colorectal cancer. In this exploratory analysis of a phase II randomized trial, high-dose vitamin D_3_ did not lead to changes of body weight, body mass index, muscle area, muscle attenuation, visceral adipose tissue area, or subcutaneous adipose tissue area, as compared with standard-dose vitamin D_3_. High-dose vitamin D_3_ did not change body composition in patients receiving chemotherapy for advanced or metastatic colorectal cancer.

**Abstract:**

Skeletal muscle and adipose tissue express the vitamin D receptor and may be a mechanism through which vitamin D supplementation slows cancer progression and reduces cancer death. In this exploratory analysis of a double-blind, multicenter, randomized phase II clinical trial, 105 patients with advanced or metastatic colorectal cancer who were receiving chemotherapy were randomized to either high-dose vitamin D_3_ (4000 IU) or standard-dose (400 IU) vitamin D_3_. Body composition was measured with abdominal computed tomography at enrollment (baseline) and after cycle 8 of chemotherapy (16 weeks). As compared with standard-dose vitamin D_3_, high-dose vitamin D_3_ did not significantly change body weight [−0.7 kg; (95% CI: −3.5, 2.0)], body mass index [−0.2 kg/m^2^; (95% CI: −1.2, 0.7)], muscle area [−1.7 cm^2^; (95% CI: −9.6, 6.3)], muscle attenuation [−0.4 HU; (95% CI: −4.2, 3.2)], visceral adipose tissue area [−7.5 cm^2^; (95% CI: −24.5, 9.6)], or subcutaneous adipose tissue area [−8.3 cm^2^; (95% CI: −35.5, 18.9)] over the first 8 cycles of chemotherapy. Among patients with advanced or metastatic colorectal cancer, the addition of high-dose vitamin D_3_, vs standard-dose vitamin D_3_, to standard chemotherapy did not result in any changes in body composition.

## 1. Introduction

More than 80% of the U.S. population has vitamin D insufficiency (e.g., 25-hydroxyvitamin D [25(OH)D] concentrations ≤30 ng/mL) [1]. Observational studies report that vitamin D insufficiency is independently associated with a higher risk of cancer death [2,3]. Meta-analyses of randomized controlled trials demonstrate that vitamin D supplementation reduces cancer death [4,5]. However, the mechanisms through which vitamin D supplementation may slow cancer progression and reduce cancer death are incompletely understood [6,7].

Skeletal muscle and adipose tissue express the vitamin D receptor [8,9,10,11]. In skeletal muscle, binding of the vitamin D receptor stimulates protein synthesis, resulting in muscle cell proliferation and growth [12,13]. Detailed reviews of these biological relationships have been reviewed elsewhere [14,15]. In adipose tissue, vitamin D and its receptor have been implicated in adipogenesis, lipid mobilization and utilization, and adipokine secretion [16,17]. This is relevant because measures of skeletal muscle and adipose tissue are prognostic of cancer progression and death in patients with various types of malignancies [18]. It is not known if the effects of vitamin D supplementation to slow cancer progression and reduce cancer death are mediated, in part, by changes in skeletal muscle and adipose tissue.

These observations provided the scientific rationale to conduct an exploratory analysis using data from the SUNSHINE trial. The SUNSHINE trial was a phase II randomized clinical trial that established the safety and preliminary efficacy of oral supplementation with high-dose vitamin D_3_ (4,000 IU) as compared with standard-dose (400 IU) vitamin D_3_ on progression-free survival in patients with advanced or metastatic colorectal cancer [19]. We hypothesized that high-dose vitamin D_3_ would increase skeletal muscle and reduce adipose tissue, as compared with standard-dose vitamin D_3_. Moreover, we hypothesized that the previously-reported improvement in progression-free survival with high-dose vitamin D_3_ would be mediated by changes in skeletal muscle and adipose tissue [19].

## 2. Results

### 2.1. Baseline Participant Characteristics

**Figure 1:**
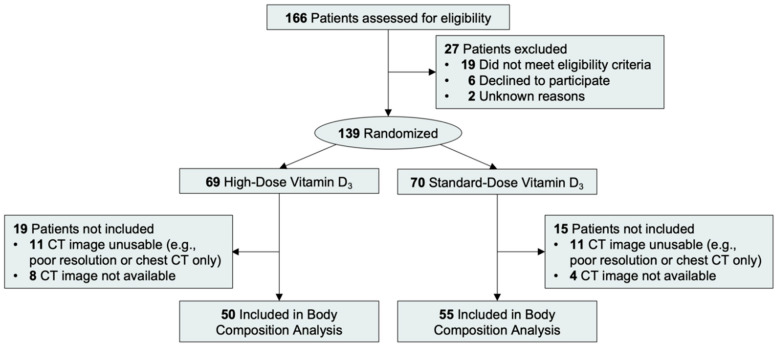
Flow of participants.

Participant recruitment was conducted from 29 March 2012 and 9 November 2016. In total, 139 participants were randomized; 105 participants were evaluable in this exploratory analysis, which did not differ between groups (*p* = 0.32); the most common reason participants were not included in this analysis was because the obtained computed tomography image needed to quantify body composition was of insufficient quality or did not include the abdominal region (Figure 1). The 105 participants included in this exploratory analysis were significantly (*p* < 0.001) more likely to have mutated (48.8% vs 23.5%) or unknown (8.6% vs 2.9%) KRAS status than the 34 participants that were not included in this analysis (Table S1). As of September 1, 2018, all participants had completed study assigned treatments. The median follow-up from randomization was 22.9 months (IQR: 11.8–34.5 months). During follow-up, we observed 86 progression-free survival events and 77 overall survival events.

Baseline participant and tumor characteristics were balanced between the treatment groups (Table 1). The 75 participants in this analysis that had both, baseline and follow-up, body composition measures did not differ on any measured baseline characteristics than the 33 participants who only had one body composition measure (Table S2). Adherence to vitamin D_3_ was high, with a median of 98% of expected capsules taken by participants in both treatment groups. As compared with participants assigned to standard-dose vitamin D_3_, those assigned to high-dose vitamin D_3_ had significantly increased concentrations of plasma 25(OH)D over the first 8 cycles of chemotherapy [20.0 ng/mL; (95% CI: 14.7, 25.2); *p* < 0.001] (Table S3).

### 2.2. Effects of Intervention on Body Composition Outcome Measures

As compared with participants assigned to standard-dose vitamin D_3_, those assigned to high-dose vitamin D_3_ supplementation did not significantly change body weight [−0.7 kg; (95% CI: −3.5, 2.0); *p* = 0.61], body mass index [−0.2 kg/m^2^; (95% CI: −1.2, 0.7); *p* = 0.63], muscle area [−1.7 cm^2^; (95% CI: −9.6, 6.3); *p* = 0.68], muscle attenuation [−0.4 HU; (95% CI: −4.2, 3.2); *p* = 0.81], visceral adipose tissue area [−7.5 cm^2^; (95% CI: −24.5, 9.6); *p* = 0.39], or subcutaneous adipose tissue area [−8.3 cm^2^; (95% CI: −35.5, 18.9); [*p* = 0.55] over the first 8 cycles of chemotherapy (Table 2). Results were similar in sensitivity analyses using maximum-likelihood regression without multiple imputation (Table S4). Nine participants experienced disease progression with the first 8 cycles of chemotherapy [2 (4.0%) in the high-dose vitamin D_3_ group and 7 (12.7%) in the standard-dose vitamin D_3_ group, *p* = 0.11]; results were similar after excluding these participants.

### 2.3. Correlation between Change in Plasma 25(OH)D and Body Composition

Among all participants, change in plasma 25(OH)D concentration from baseline to cycle 8 was not significantly associated with change in body weight [*r* = −0.24; (95% CI: −0.46, 0.01); *p* = 0.054], body mass index [*r* = −0.23; (95% CI: −0.45, 0.02); *p* = 0.067], muscle area [*r* = −0.17; (95% CI: −0.41, 0.09); *p* = 0.19], muscle attenuation [*r* = 0.16; (95% CI: −0.10, 0.41); *p* = 0.21], visceral adipose tissue area [*r* = −0.05; (95% CI: −0.30, 0.21); *p* = 0.71], and subcutaneous adipose tissue area [*r* = 0.01; (95% CI: −0.26, 0.26); *p* = 0.99].

### 2.4. Mediation Effect of Body Composition on Vitamin D_3_ and Progression-Free Survival

In the subgroup of 105 participants included this exploratory analysis, randomization to high-dose vitamin D_3_ was associated with a lower risk of disease progression or death as compared with low-dose vitamin D_3_ [HR: 0.67; (95% CI: 0.42, 1.07)]; the magnitude of risk reduction was similar to that observed in the full analysis set of 139 participants as previously reported [HR: 0.64; (95% CI: 0.0–0.90)] [19]. Change in body weight [HR: 0.69; 95% CI: 0.40, 1.18)], body mass index [HR: 0.69; (95% CI: 0.40, 1.17)], muscle area [HR: 0.62; (95% CI: 0.35, 1.11)], muscle attenuation [HR: 0.73; (95% CI: 0.42, 1.25)], visceral adipose tissue area [HR: 0.77; (95% CI: 0.44, 1.36)], and subcutaneous adipose tissue area [HR: 0.76; (95% CI: 0.44, 1.33)] over the first 8 cycles of chemotherapy did not mediate the association between randomized group and progression-free survival (Table 3).

### 2.5. Prognostic Effect of Body Composition on Progression-Free and Overall Survival

In restricted cubic spline analysis, no baseline body composition measures were significantly associated with progression-free survival (Figure S1); baseline muscle area (nonlinear *p* = 0.026) and visceral adipose tissue area (nonlinear *p* = 0.01) were significantly associated with overall survival (Figure S2). Change in muscle attenuation from baseline to cycle 8 (nonlinear *p* = 0.002) was significantly associated with progression-free survival (Figure S3); no change in body composition measures from baseline to cycle 8 were significantly associated with overall survival (Figure S4).

## 3. Discussion

In this exploratory analysis of a phase II trial, high-dose vitamin D_3_ vs standard-dose vitamin D_3_ did not significantly change skeletal muscle and adipose tissue among patients with metastatic colorectal cancer receiving standard chemotherapy. Among both randomized groups, change in plasma 25(OH)D concentration did not correlate with changes in body composition. Change in skeletal muscle and adipose tissues did not mediate the effect of high-dose vitamin D_3_ on progression-free survival in this population. Among both randomized groups, baseline muscle area and visceral adipose tissue area were associated with overall survival and change in muscle attenuation from baseline to cycle 8 was associated with progression-free survival. These exploratory findings help to clarify the potential mechanisms through which vitamin D supplementation may slow cancer progression and reduce cancer death.

At the time of diagnosis, ≥80% of patients with advanced or metastatic colorectal cancer have 25(OH)D concentrations ≤30 ng/mL [20]. A prospective analysis of 1043 patients with metastatic colorectal cancer who participated in a randomized phase III clinical trial of first-line chemotherapy plus biologic therapy demonstrated that patients with plasma 25(OH)D concentrations ≥20 ng/mL had a 19% reduced risk of disease progression [HR: 0.81; (95% CI: 0.66–1.00)] and a 30% reduced risk of death [HR: 0.70; (95% CI: 0.56–0.86)], as compared with plasma 25(OH)D concentrations <10 ng/mL [21]. A meta-analysis of 11 observational studies that included 7718 patients with stage I-IV colorectal cancer demonstrated that 25(OH)D was independently and inversely associated with cancer-specific and overall survival [22].

We hypothesized that one of the mechanisms by which vitamin D_3_ supplementation exerts anticancer effects is through its impact on body composition. Our hypothesis was founded on the convergence of several lines of evidence. Skeletal muscle and adipose tissue express the vitamin D receptor [8,9,10,11]. In cross-sectional studies, concentrations of 25(OH)D positively correlate with skeletal muscle and negatively correlate with visceral and subcutaneous adipose tissue [23,24]. Skeletal muscle and adiposity are independent prognostic factors in patients with colorectal cancer [25,26,27]. The findings from this exploratory analysis, however, are not consistent with this hypothesis. Our observations support the results of several meta-analyses in various populations that vitamin D supplementation does not substantively change body composition [28,29,30].

There are several limitations to this trial. The main limitation is that this was an unplanned, exploratory, post hoc analysis and the findings, although null, should be interpreted conservatively. The relatively modest sample size may have limited our ability to detect small, but potentially clinically meaningful effects of vitamin D_3_ supplementation on body composition outcomes. The current sample size provided sufficient statistical power to detect moderate to large treatment effects. The racially and geographically homogeneous sample also limited our ability to detect treatment effects in participant subgroups. The intervention duration was 16 weeks (8 cycles of mFOLFOX6 chemotherapy), which limits our ability to understand the benefits of vitamin D_3_ supplementation on body composition over longer time horizons. The intervention did not include other supplements that may enhance the absorption of vitamin D, such as calcium, magnesium, and marine n-3 fatty acids. The study population was not recruited on the basis of having unfavorable body composition at baseline, which limits our understanding of treatment effect in patients with low muscle or excess adiposity at baseline.

There are several strengths to this trial. The randomized double-blind design allowed for a direct comparison of treatment effect of high-dose vitamin D_3_ on body composition outcomes. Study participants were recruited from both academic and community-based cancer centers. Based on changes in plasma 25(OH)D concentrations, there was high supplement adherence, and no evidence of control group crossover, despite availability of vitamin D_3_ supplements over the counter to patients. Body composition was ascertained using computed tomography, a gold-standard modality for muscle and adipose tissue measurement [31], by staff who were blinded to randomized group assignment.

## 4. Materials and Methods

### 4.1. Study Design

This study was a double-blind, multicenter, randomized phase II clinical trial. The study was conducted at 11 academic and community cancer centers across the United States. The study was conducted in accordance with Good Clinical Practice and the ethical principles originating in the Declaration of Helsinki. The protocol and informed consent document were approved by the institutional review board for each study site (coordinating center, Dana-Farber Cancer Institute, IRB Protocol 11-436; approved 12/27/2011). An independent data and safety monitoring board provided oversight of the study. All participants provided written informed consent prior to completing any study-related activities. The study was registered on ClinicalTrials.gov as NCT01516216. The detailed study protocol is published [19].

### 4.2. Participants

Patients were eligible if they had pathologically confirmed, unresectable locally advanced or metastatic adenocarcinoma of the colon or rectum with measurable disease per the Response Evaluation Criteria in Solid Tumors (RECIST) guidelines version 1.1 [32]. Patients were eligible if the last dose of prior chemotherapy or chemoradiotherapy was ≥12 months before study enrollment. Eligible patients had an Eastern Cooperative Group (ECOG) performance status of 0–1, with adequate organ function, and no evidence of hypercalcemia or conditions that may increase the risk of hypercalcemia (e.g., hyperparathyroidism). Patients were ineligible if they were taking ≥2000 IU daily of vitamin D_3_, had symptomatic genitourinary stones within the past year, or were taking thiazide diuretics.

### 4.3. Randomization and Blinding

Participants were randomly assigned by the trial statistician in a 1:1 ratio to high-dose vitamin D_3_ or standard-dose vitamin D_3_ (described in detail below) using a computerized block randomization procedure with a block size of two. The trial statistician and research pharmacist were not blinded to treatment assignment. Study participants and treating physicians were blinded to treatment assignment.

### 4.4. Intervention

All study participants received chemotherapy with the mFOLFOX6 regimen, plus bevacizumab, administered every 2 weeks (1 cycle = 2 weeks) [33]. Bevacizumab could be omitted during cycle 1 and commenced with cycle 2, per treating physician discretion.

Vitamin D_3_ capsules and placebos were identical in appearance (Pharmavite, LLC, West Hills, CA, USA). After randomization, participants were instructed to cease consumption of all supplements containing vitamin D and calcium outside of the study intervention. The high-dose vitamin D_3_ group received an initial daily dose of 8000 IU of vitamin D_3_ (two 4000 IU capsules) for cycle 1, and 4000 IU per day for all subsequent cycles. The standard-dose group received 400 IU daily during all cycles (one 400 IU capsule plus one placebo capsule during cycle 1 to maintain blinding). Adherence to vitamin D_3_ was monitored using participant diaries and pill bottle reconciliation. Plasma 25(OH)D concentrations were quantified using a radioimmunoassay (DiaSorin, Inc., Saluggia, Italy). Participants continued to receive the study intervention until disease progression, intolerable toxicity, or decision to discontinue treatment.

### 4.5. Body Composition Outcome Measures

Height (meters) and weight (kilograms) were measured by trained medical assistants. Body mass index was calculated as kilograms of body weight per square meter of height (kg/m^2^). Body composition was measured using computed tomography (CT) images that were obtained with standard clinical contrast-enhanced protocols using slice-O-matic software (V4.3, TomoVision, Montreal, QC, Canada). A single slice transverse image at the third lumbar vertebra was used because tissue cross-sectional areas at this lumbar region are correlated with whole-body tissue volume [34,35]. Tissues were demarcated with a semiautomated procedure using Hounsfield Unit thresholds of −29 to 150 for muscle tissue (including all paraspinal and abdominal wall muscles), −150 to −50 for visceral adipose tissue, and −190 to −30 for subcutaneous adipose tissue. Cross-sectional areas were calculated for each tissue compartment by summing tissue pixels and multiplying by the pixel surface area. Muscle radiodensity quantified the average radiation attenuation rate as a radiologic measure of the extent of lipid contained within muscle [36]. Images were analyzed by trained staff who were blinded to study hypothesis, trial design, and image order (baseline vs. restaging follow-up). Coefficients of variation were 0.5% for muscle (individual reader range: 0.5–1.1%), 0.7% (0.4–1.0%) for visceral adipose tissue area, and 0.4% (0.2–0.5%) for subcutaneous adipose area [37]. Final data verification was performed by a board-certified radiologist who was blinded to randomized group assignment (M.H.R.). Body composition was analyzed at baseline (pre-treatment) and at the second tumor restaging (cycle 8 of chemotherapy = 16 weeks of randomized study treatment).

### 4.6. Other Measures

Data for participant characteristics including age, sex, race and ethnicity, ECOG performance status, primary tumor location, primary tumor resection status, receipt of prior cancer-directed therapy, number of metastatic sites, carcinoembryonic antigen (CEA) concentration, and tumor mutational profile—including microsatellite instability, *KRAS*, *NRAS*, and *BRAF V600E* status—were obtained from a combination of participant self-report, physician assessment, and the medical record.

### 4.7. Statistical Analysis

The sample size was selected to provide sufficient statistical power to detect change in the primary endpoint of progression-free survival [19]. Measures of body composition were analyzed as exploratory study outcomes. Descriptive statistics presented for baseline variables include counts with proportions for categorical variables and medians with interquartile (25–75%) ranges for continuous variables. Categorical baseline characteristics were compared using the Fisher’s exact or χ^2^ tests, and continuous baseline characteristics were compared using the Kruskal–Wallis or *t*-tests.

All analyses adhered to the modified intention-to-treat principle. The primary modeling strategy evaluated the treatment policy estimand (i.e., the treatment effect regardless of adherence or discontinuation) quantified using a generalized linear model for repeated measures with missing data imputed by a pattern mixture model with multiple imputation [38,39]. The secondary modeling strategy evaluated the trial product estimand (i.e., the treatment effect assuming all patients remained on trial) quantified using a mixed model for repeated measures with observed data (e.g., no imputation) [38]. The baseline value of the dependent variable was included as a covariate in the regression models [40]. Treatment effects were estimated as the group-by-time interaction with least-square means ± standard error or corresponding 95% confidence intervals. Model fit was assessed using graphical and numeric techniques. Sensitivity analyses excluded participants who experienced tumor progression within the first 8 cycles of chemotherapy. The Pearson correlation coefficient with bootstrapped 95% confidence intervals were used to quantify the strength of the association between change in plasma 25(OH)D and body composition [41].

The degree to which change in skeletal muscle and adipose tissue mediate the previously-reported treatment effect on progression-free survival was estimated using techniques for continuous mediators and time-to-event outcomes [42]. Additional analyses that consolidated the two randomized groups were conducted to quantify the association between body composition with progression-free survival and overall survival outcomes. Multivariable-adjusted Cox proportional hazards models were used to estimate hazard ratios and 95% CIs with restricted cubic splines [43]. Models were adjusted a priori for age, sex, race/ethnicity, ECOG performance status, and the number of metastatic sites [19]. The proportionality of hazards assumption was examined with visual inspection of log–log plots and tested in a regression model of the scaled Schoenfeld residuals on time [44].

## 5. Conclusions

Among patients with advanced or metastatic colorectal cancer, the addition of high-dose vitamin D_3_, vs standard-dose vitamin D_3_, to standard chemotherapy did not result in any differences in body composition. The findings from this exploratory study indicate that the benefits of vitamin D_3_ on reducing cancer progression and death are unlikely to be mediated by changes in body composition. A multicenter, double-blind, randomized phase III trial is currently underway to evaluate the efficacy of high-dose vs. standard-dose vitamin D_3_ on progression-free survival in 400 patients with metastatic colorectal cancer, and the correlative studies embedded into this trial will offer unprecedented insight into mechanisms of treatment benefit [45].

## Figures and Tables

**Table 1:** Baseline characteristics of sub-study participants by randomized group.

Characteristic	High-Dose Vitamin D_3_ (*n* = 50)	Standard-Dose Vitamin D_3_ (*n* = 55)
Age, median (IQR), y	54.2 (46.8–65.3)	55.5 (49.2–64.7)
Sex, No. (%)		
Male	32 (64.0)	27 (49.1)
Female	18 (36.0)	28 (50.9)
Race, Ethnicity, No. (%)		
White	38 (76.0)	43 (78.2)
Black	2 (4.0)	5 (9.1)
Asian	0 (0)	0 (0)
>1 Race	0 (0.0)	1 (1.8)
Other	10 (20.0)	6 (10.9)
ECOG Performance Status, No. (%)		
0	21 (42.0)	32 (58.2)
1	29 (58.0)	23 (41.8)
Primary Tumor Location, No. (%)		
Right Colon	12 (24.0)	15 (27.3)
Transverse Colon	3 (6.0)	7 (12.7)
Left Colon, Rectum	35 (70.0)	33 (60.0)
Primary Tumor Resected, No. (%)	20 (40.0)	17 (30.9)
No. of Metastatic Sites, mean (SD)	2.0 (0.93)	1.9 (0.91)
Carcinoembryonic Antigen *, median (IQR), ng/mL	64.8 (4.5–565.6)	91.9 (5.5–393.5)
Microsatellite Instability Status, No. (%)		
High	1 (2.0)	4 (7.3)
Stable	42 (84.0)	35 (63.6)
Unknown	7 (14.0)	16 (29.1)
*KRAS* Mutation Status, No. (%)		
Wild Type	26 (52.0)	24 (43.6)
Mutated	22 (44.0)	24 (43.6)
Unknown	2 (4.0)	7 (12.7)
*NRAS* Mutation Status, No. (%)		
Wild Type	29 (58.0)	30 (54.5)
Mutated	0 (0.0)	2 (3.6)
Unknown	21 (42.0)	23 (41.8)
*BRAF V600E* Mutation Status, No. (%)		
Wild Type	31 (62.0)	30 (54.5)
Mutated	3 (6.0)	7 (12.7)
Unknown	16 (32.0)	18 (32.7)

* Missing for 1 participant.

**Table 2:** Effects of vitamin D_3_ supplementation on change in body composition outcomes using multiple imputation analysis.

Outcome & Group	Baseline [LS Mean (SE)]	Follow-Up [LS Mean (SE)]	Δ Baseline to Follow-Up (LS Mean, 95% CI)	Δ Between Group (LS Mean, 95% CI)	*p*
Body Weight, kg					
High-Dose Vitamin D_3_	82.0 (3.1)	81.0 (3.3)	−1.0 (−2.9, 0.9)	−0.7 (−3.5, 2.0)	0.61
Standard-Dose Vitamin D_3_	76.8 (3.2)	76.5 (3.4)	−0.3 (−2.3, 1.7)	―	
Body Mass Index, kg/m^2^					
High-Dose Vitamin D_3_	28.7 (0.99)	28.4 (1.03)	−0.3 (−1.0, 0.3)	−0.2 (−1.2, 0.7)	0.63
Standard-Dose Vitamin D_3_	27.2 (1.03)	27.1 (1.07)	−0.1 (−0.8, 0.6)	―	
Muscle Area, cm^2^					
High-Dose Vitamin D_3_	139.3 (4.6)	135.4 (4.9)	−3.9 (−8.8, 1.0)	−1.7 (−9.6, 6.3)	0.68
Standard-Dose Vitamin D_3_	133.5 (4.8)	131.3 (5.3)	−2.3 (−7.9, 3.4)	―	
Muscle Attenuation, HU					
High-Dose Vitamin D_3_	34.9 (1.47)	35.0 (1.74)	0.1 (−2.4, 2.6)	−0.4 (−4.2, 3.2)	0.81
Standard-Dose Vitamin D_3_	38.0 (1.52)	38.6 (1.73)	0.6 (−2.2, 3.4)	―	
Visceral Adipose Tissue Area, cm^2^					
High-Dose Vitamin D_3_	130.8 (15.3)	128.0 (15.3)	−2.8 (−14.7, 9.2)	−7.5 (−24.5, 9.6)	0.39
Standard-Dose Vitamin D_3_	111.5 (15.8)	116.1 (16.0)	4.7 (−7.9, 17.3)	―	
Subcutaneous Adipose Tissue Area, cm^2^					
High-Dose Vitamin D_3_	230.5 (20.1)	226.0 (22.1)	−4.5 (−24, 15.1)	−8.3 (−35.5, 18.9)	0.55
Standard-Dose Vitamin D_3_	207.6 (20.6)	211.5 (22.6)	3.8 (−15.2, 22.9)	―	

All results were from a regression model for repeated measurements that was adjusted for age, number of metastatic sites, sex, race, and ECOG performance status.

**Table 3:** Change in effect of vitamin D_3_ supplementation randomized group on progression-free survival before and after adjustment for change in body composition.

Before Adjustment Hazard Ratio (95% CI)	Hypothesized Mediator	After Adjustment Hazard Ratio (95% CI)
0.67 (0.42, 1.07)		
	Δ Body Weight	0.69 (0.40, 1.18)
	Δ Body Mass Index	0.69 (0.40, 1.17)
	Δ Muscle Area	0.62 (0.35, 1.11)
	Δ Muscle Attenuation	0.73 (0.42, 1.25)
	Δ Visceral Adipose Tissue Area	0.77 (0.44, 1.36)
	Δ Subcutaneous Adipose Tissue Area	0.76 (0.44, 1.33)

Hazard ratios compare high-dose vitamin D_3_ supplementation with standard-dose vitamin D_3_ supplementation and were estimated from a Cox proportional hazards model that was adjusted for age, number of metastatic sites, sex, race, and ECOG performance status.

## Supplementary Materials

The following are available online at https://www.mdpi.com/2072-6694/12/11/3451/s1, Figure S1: Association of baseline body composition with progression-free survival (PFS), Figure S2: Association of baseline body composition with overall survival (OS), Figure S3: Association of change in body composition from baseline to follow-up with progression-free survival (PFS), Figure S4: Association of change in body composition from baseline to follow-up with overall survival (OS), Table S1: Comparison of baseline characteristics of sub-study participants compared to non-participants, Table S2: Comparison of baseline characteristics of sub-study participants with baseline and follow-up body composition measures compared to only baseline body composition measures, Table S3: Change in of vitamin D3 supplementation on plasma 25-hydroxyvitamin D concentrations among body composition sub-study participants, Table S4: Effects of vitamin D_3_ supplementation on change in body composition outcomes using maximum likelihood regression without multiple imputation.
