# Supplementary material for: Effect of High-Dose vs Standard-Dose Vitamin D3 Supplementation on Body Composition among Patients with Advanced or Metastatic Colorectal Cancer: A Randomized Trial
Source: Cancers (Basel). 2020 Nov 20;12(11):3451. doi: 10.3390/cancers12113451 (PMC7699725; doi:10.3390/cancers12113451)
Supplement: Supplementary file 1 [file cancers-12-03451-s001.pdf]

Supplementary Materials: Effect of High-Dose VS Standard-Dose Vitamin D3 Supplementation on Body Composition Among Patients with Advanced or Metastatic Colorectal Cancer: A Randomized Trial

Justin C. Brown, Michael H. Rosenthal, Chao Ma, Sui Zhang, Halla S. Nimeiri, Nadine J. McCleary, Thomas A. Abrams, Matthew B. Yurgelun, James M. Cleary, Douglas A. Rubinson, Deborah Schrag, Andrea J. Bullock, Jill Allen, Dan Zuckerman, Emily Chan, Jennifer A. Chan, Brian Wolpin, Michael Constantine, Douglas J. Weckstein, Meredith A. Faggen, Christian A. Thomas, Chryssanthi Kournioti, Chen Yuan, Hui Zheng, Bruce W. Hollis, Charles S. Fuchs, Kimmie Ng and Jeffrey A. Meyerhardt

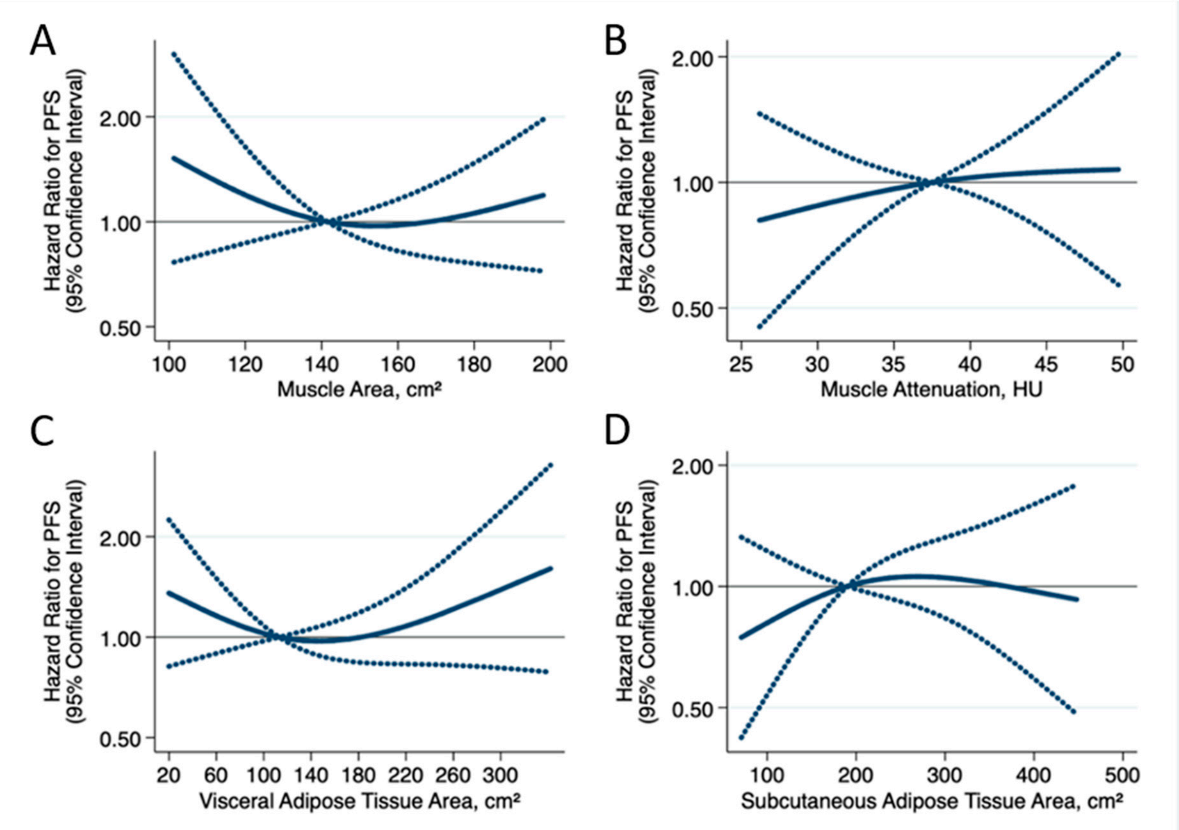

Figure S1. Association of baseline body composition with progression-free survival (PFS), by (**A**) muscle area; (**B**) muscle attenuation; (**C**) visceral adipose tissue area; and (**D**) subcutaneous adipose tissue area. Solid line represents the point estimate of the hazard ratio and dotted lines represent the corresponding 95% confidence intervals. Estimates are multivariable adjusted for age, number of metastatic sites, sex, race, and ECOG performance status. Note differential *x*- and *y*-axis scaling between graphs.

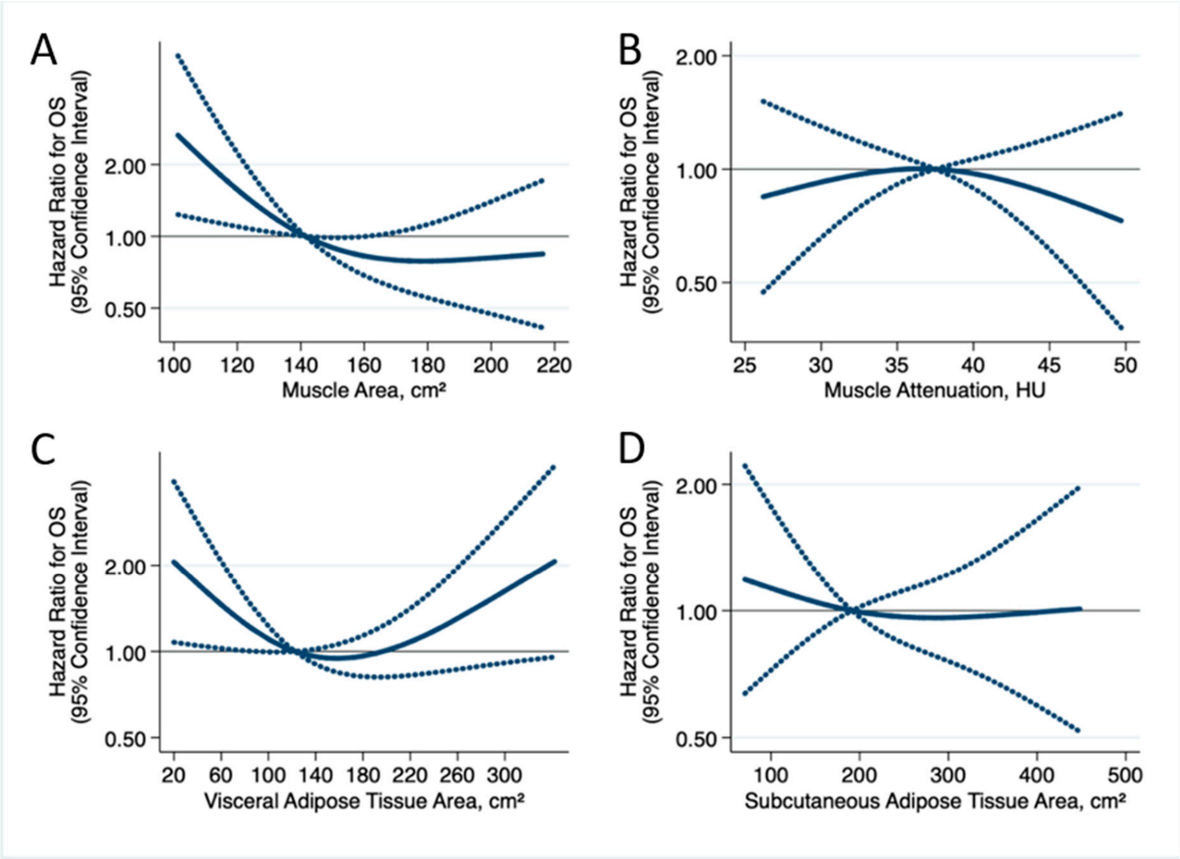

Figure S2. Association of baseline body composition with overall survival (OS), by (**A**) muscle area; (**B**) muscle attenuation; (**C**) visceral adipose tissue area; and (**D**) subcutaneous adipose tissue area. Solid line represents the point estimate of the hazard ratio and dotted lines represent the corresponding 95% confidence intervals. Estimates are multivariable adjusted for age, number of metastatic sites, sex, race, and ECOG performance status. Note differential *x*- and *y*-axis scaling between graphs.

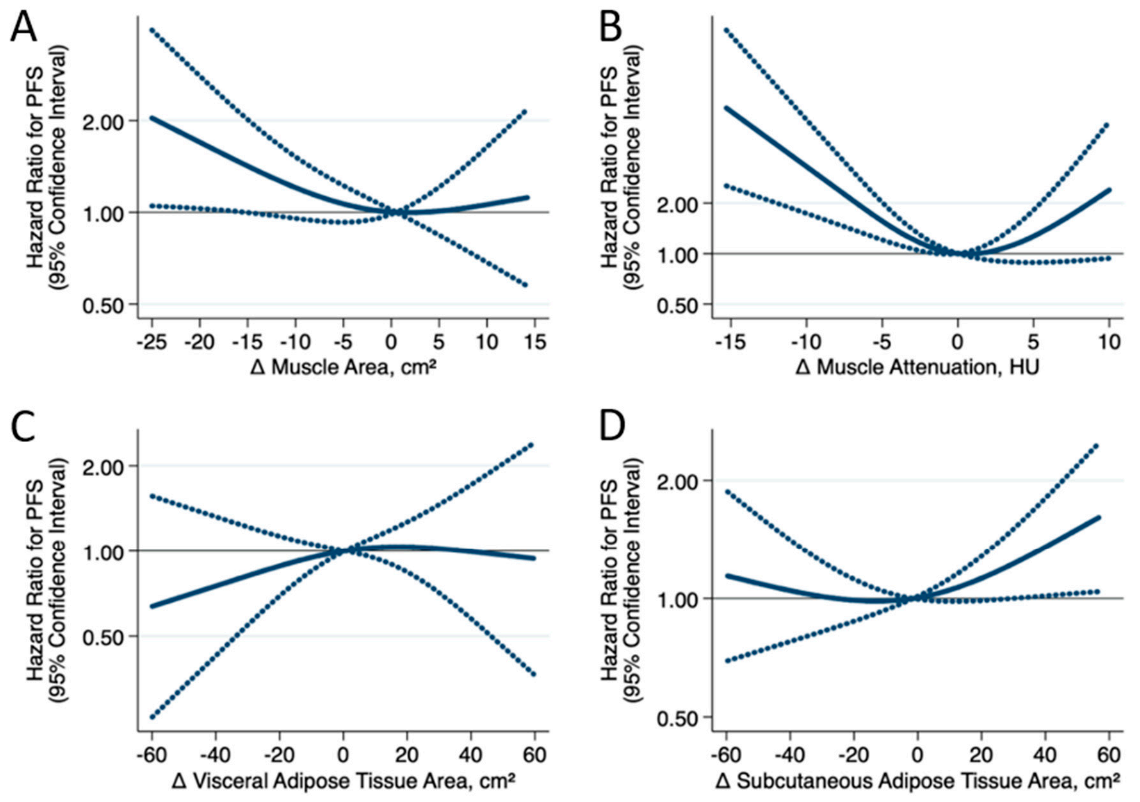

Figure S3. Association of change in body composition from baseline to follow-up with progression-free survival (PFS), by (**A**) muscle area; (**B**) muscle attenuation; (**C**) visceral adipose tissue area; and (**D**) subcutaneous adipose tissue area. Solid line represents the point estimate of the hazard ratio and dotted lines represent the corresponding 95% confidence intervals. Estimates are multivariable adjusted for age, number of metastatic sites, sex, race, and ECOG performance status. Note differential *x*- and *y*-axis scaling between graphs.

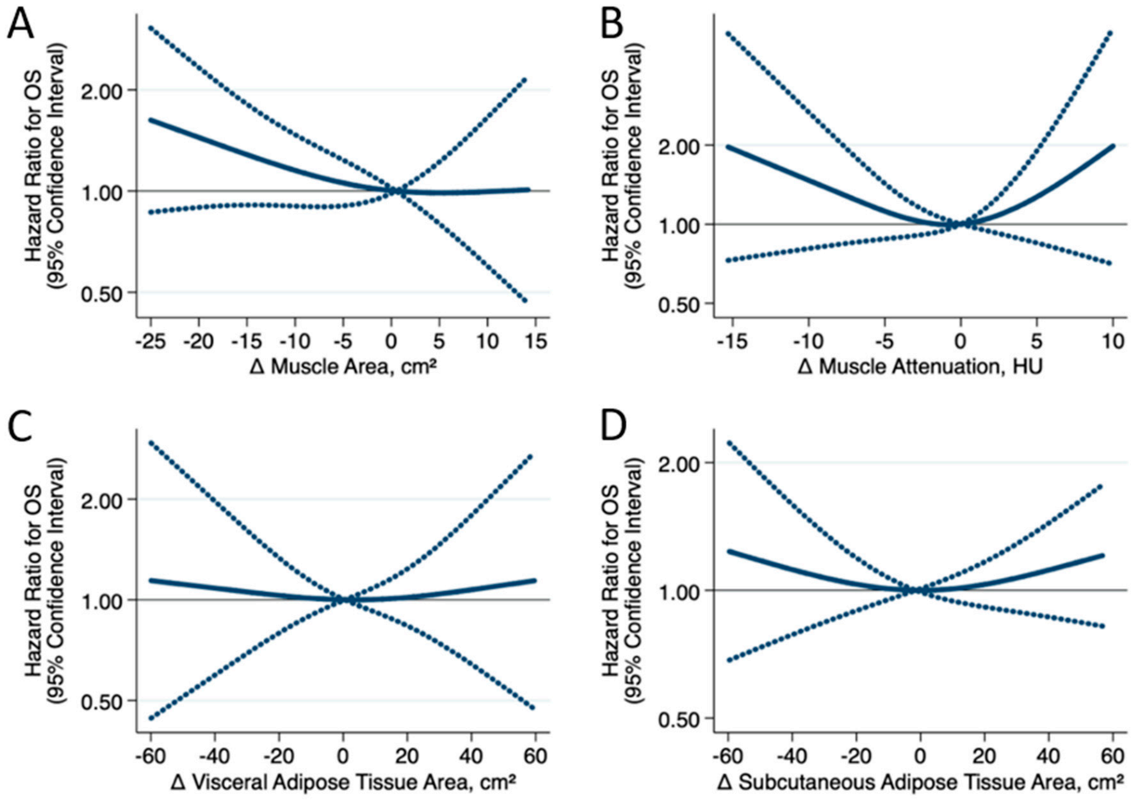

Figure S4. Association of change in body composition from baseline to follow-up with overall survival (OS), by (**A**) muscle area; (**B**) muscle attenuation; (**C**) visceral adipose tissue area; and (**D**) subcutaneous adipose tissue area. Solid line represents the point estimate of the hazard ratio and dotted lines represent the corresponding 95% confidence intervals. Estimates are multivariable adjusted for age, number of metastatic sites, sex, race, and ECOG performance status. Note differential *x*- and *y*-axis scaling between graphs.

Table S1. Comparison of baseline characteristics of sub-study participants compared to non-participants.

Characteristic	Sub-Study Participants (n = 105)	Non-Participants (n = 34)
Age, median (IQR), y	54.6 (47.7–64.9)	54.7 (48.5–61.4)
Sex, No. (%)		
Male	59 (56.2)	20 (58.8)
Female	46 (43.8)	14 (41.2)
Race, Ethnicity, No. (%)		
White	81 (77.1)	26 (76.5)
Black	7 (6.7)	3 (8.8)
Asian	0 (0.0)	1 (2.9)
>1 Race	1 (0.95)	2 (5.9)
Other	16 (15.2)	2 (5.9)
ECOG Performance Status, No. (%)		
0	53 (50.5)	16 (47.1)
1	52 (49.5)	18 (52.9)
Primary Tumor Location, No. (%)		
Right Colon	27 (25.7)	8 (23.5)
Transverse Colon	10 (9.5)	2 (5.9)
Left Colon, Rectum	68 (64.8)	24 (70.6)
Primary Tumor Resected, No. (%)	37 (35.2)	10 (29.4)
No. of Metastatic Sites, mean (SD)	1.9 (0.92)	1.7 (0.84)
Carcinoembryonic Antigen *, median (IQR), ng/mL	66.0 (5.3-406.9)	38.7 (11.4-142.0)
Microsatellite Instability Status, No. (%)		
High	5 (4.8)	1 (2.9)
Stable	77 (73.3)	27 (79.4)
Unknown	23 (21.9)	6 (17.6)
KRAS Mutation Status, No. (%)		
Wild Type	50 (47.6)	25 (73.5)
Mutated	46 (43.8)	8 (23.5)
Unknown	9 (8.6)	1 (2.9)
NRAS Mutation Status, No. (%)		
Wild Type	59 (56.2)	20 (58.8)
Mutated	2 (1.9)	2 (5.9)
Unknown	44 (41.9)	12 (35.3)
BRAF V600E Mutation Status, No. (%)		
Wild Type	61 (58.1)	16 (47.1)

Mutated	10 (9.5)	4 (11.8)
Unknown	34 (32.4)	14 (41.2)
Randomized Group, No. (%)		
High-Dose Vitamin D_3	50 (47.6)	19 (55.9)
Standard-Dose Vitamin D_3	55 (52.4)	15 (44.1)
Body Weight, median (IQR), kg	77.6 (64.6–89.7)	76.9 (63.1–96.6)
Body Mass Index, median (IQR), kg/m^2	26.3 (23.4–29.9)	25.7 (20.9–32.5)
Plasma 25(OH)D †, median (IQR), ng/mL	16.7 (11.5–22.2)	19.6 (13.4–25.5)

* Missing for 1 participant. † Missing for 15 participants.

Table S2. Comparison of baseline characteristics of sub-study participants with baseline and follow-up body composition measures compared to only baseline body composition measures.

Characteristic	Baseline & Follow-Up (*n* = 75)	Baseline Only (*n* = 21)	Follow-Up Only (*n* = 9)
Age, median (IQR), y	53.5 (46.8–65.0)	60.3 (53.8–65.5)	52.3 (50.1–58.9)
Sex, No. (%)			
Male	39 (52.0)	16 (76.2)	4 (44.4)
Female	36 (48.0)	5 (23.8)	5 (55.6)
Race, Ethnicity, No. (%)			
White	61 (81.3)	15 (71.4)	5 (55.6)
Black	4 (5.3)	1 (4.8)	2 (22.2)
Asian	0 (0.0)	0 (0.0)	0 (0.0)
>1 Race	0 (0.0)	1 (4.8)	0 (0.0)
Other	10 (13.3)	4 (19.0)	2 (22.2)
ECOG Performance Status, No. (%)			
0	35 (46.7)	11 (52.4)	7 (77.8)
1	40 (53.3)	10 (47.6)	2 (22.2)
Primary Tumor Location, No. (%)			
Right Colon	21 (28.0)	5 (23.8)	1 (11.1)
Transverse Colon	5 (6.7)	4 (19.0)	1 (11.1)
Left Colon, Rectum	49 (65.3)	12 (57.1)	7 (77.8)
Primary Tumor Resected, No. (%)	30 (40.0)	7 (33.3)	0 (0.0)
No. of Metastatic Sites, mean (SD)	2.0 (0.93)	1.6 (0.74)	2.0 (1.1)
Carcinoembryonic Antigen, median (IQR), ng/mL	133.0 (5.0–576.8)	30.6 (5.6–333.5)	57.9 (26.0–101.0)
Microsatellite Instability Status, No. (%)			
High	3 (4.0)	2 (9.5)	0 (0.0)
Stable	57 (76.0)	12 (57.1)	8 (88.9)

Unknown	15 (20.0)	7 (33.3)	1 (11.1)
KRAS Mutation Status, No. (%)			
Wild Type	36 (48.0)	8 (38.1)	6 (66.7)
Mutated	35 (46.7)	8 (38.1)	3 (33.3)
Unknown	4 (5.3)	5 (23.8)	0 (0.0)
NRAS Mutation Status, No. (%)			
Wild Type	45 (60.0)	8 (38.1)	6 (66.7)
Mutated	2 (2.7)	0 (0.0)	0 (0.0)
Unknown	28 (37.3)	13 (61.9)	3 (33.3)
BRAF V600E Mutation Status, No. (%)			
Wild Type	48 (64.0)	7 (33.3)	6 (66.7)
Mutated	6 (8.0)	3 (14.3)	1 (11.1)
Unknown	21 (28.0)	11 (52.4)	2 (22.2)
Body Weight, median (IQR), kg	77.6 (64.1–92.1)	80 (59.9–90.2)	76.2 (65.7–82.1)
Body Mass Index, median (IQR), kg/m^2	26.3 (23.4–30.5)	26.4 (22.2–29.0)	26.3 (24.2–28.6)
Muscle Area, median (IQR), cm^2	141.5 (109.4–177.5)	137.4 (113.0–169.6)	—
Muscle Attenuation, median (IQR), HU	37.1 (30.6–44.8)	37.7 (33.0–40.4)	—
Visceral Adipose Tissue, median (IQR), cm^2	111.3 (47.8–199.6)	143.4 (64.8–230.9)	—
Subcutaneous Adipose Tissue, median (IQR), cm^2	199.2 (125.8–282.8)	152.3 (113.6–235.4)	—
Randomized Group, No. (%)			
High-Dose Vitamin D_3	41 (54.7)	6 (28.6)	3 (33.3)
Standard-Dose Vitamin D_3	34 (45.3)	15 (71.4)	6 (66.7)
Plasma 25(OH)D, median (IQR), ng/mL	17.2 (11.4–22.3)	19.5 (14.2–22.4)	16.5 (9.7–16.5)

Table S3. Change in of vitamin D_3 supplementation on plasma 25-hydroxyvitamin D concentrations among body composition sub-study participants.

**Plasma 25(OH)-D, ng/mL**	**Baseline [LS Mean (SE)]**	**Follow-Up [LS Mean (SE)]**	**Δ Baseline to Follow-Up (LS Mean, 95% CI)**	**Δ Between Group (LS Mean, 95% CI)**	***p***
High-Dose Vitamin D_3	15.1 (1.2)	35.8 (1.6)	20.7 (17.1, 24.4)	20.0 (14.7, 25.2)	<0.001
Standard-Dose Vitamin D_3	18.2 (1.2)	18.9 (1.7)	0.8 (−3.0, 4.6)	—	

Table S4. Effects of vitamin D_3 supplementation on change in body composition outcomes using maximum likelihood regression without multiple imputation.

Outcome & Group	Baseline [LS Mean (SE)]	Follow-Up [LS Mean (SE)]	Δ Baseline to Follow-Up (LS Mean, 95% CI)	Δ Between Group (LS Mean, 95% CI)	*p*
Body Weight, kg					
High-Dose Vitamin D_3	82.3 (3.19)	81.5 (3.23)	−0.8 (−2.6, 0.8)	−1.0 (−3.5, 1.6)	0.45
Standard-Dose Vitamin D_3	76.9 (3.3)	77.0 (3.36)	0.1 (−1.7, 2.0)	—	
Body Mass Index, kg/m^2					
High-Dose Vitamin D_3	28.8 (1.01)	28.5 (1.02)	−0.3 (−0.9, 0.3)	−0.3 (1.1, 0.6)	0.51
Standard-Dose Vitamin D_3	27.2 (1.04)	27.2 (1.06)	−0.01 (−0.6, 0.6)	—	
Muscle Area, cm^2					
High-Dose Vitamin D_3	140.1 (4.6)	136.3 (4.7)	−3.8 (−7.9, 0.4)	−1.4 (−7.5, 4.8)	0.66
Standard-Dose Vitamin D_3	134.1 (4.8)	131.8 (4.9)	−2.4 (−6.9, 2.1)	—	
Muscle Attenuation, HU					
High-Dose Vitamin D_3	35.6 (1.39)	36.0 (1.49)	0.4 (−1.7, 2.5)	0.8 (−2.4, 3.9)	0.63
Standard-Dose Vitamin D_3	38.6 (1.44)	38.2 (1.6)	−0.4 (−2.7, 1.9)	—	
Visceral Adipose Tissue Area, cm^2					
High-Dose Vitamin D_3	127.3 (15.3)	126.7 (14.9)	−0.6 (−10.5, 9.2)	−8.8 (−23.3, 5.7)	0.23
Standard-Dose Vitamin D_3	109.9 (15.9)	118.1 (15.7)	8.2 (−2.5, 18.9)	—	
Subcutaneous Adipose Tissue Area, cm^2					
High-Dose Vitamin D_3	231.9 (20.1)	228.3 (21.4)	−3.6 (−20.1, 12.9)	−8.8 (−33.2, 15.6)	0.48
Standard-Dose Vitamin D_3	208.2 (20.8)	213.3 (22.3)	5.2 (−12.8, 23.1)	—	

All results were from a linear mixed model for repeated measurements that was adjusted for age, number of metastatic sites, sex, race, and ECOG performance status.

Publisher's Note: MDPI stays neutral with regard to jurisdictional claims in published maps and institutional affiliations.

© 2020 by the authors. Licensee MDPI, Basel, Switzerland. This article is an open access article distributed under the terms and conditions of the Creative Commons Attribution (CC BY) license (http://creativecommons.org/licenses/by/4.0/).
